# The Renal Arterial Resistive Index and Stage of Chronic Kidney Disease in Patients with Renal Allograft

**DOI:** 10.1371/journal.pone.0051772

**Published:** 2012-12-14

**Authors:** Stine O. Winther, Helle C. Thiesson, Lene N. Poulsen, Mahtab Chehri, Hanne Agerskov, Martin Tepel

**Affiliations:** 1 Department of Nephrology, Odense University Hospital, Odense, Denmark; 2 Institute of Molecular Medicine, Cardiovascular and Renal Research, University of Southern Denmark, Odense, Denmark; University of São Paulo School of Medicine, Brazil

## Abstract

**Objective:**

The study investigated the optimal threshold value of renal arterial resistive index as assessed by Doppler ultrasonography determining chronic kidney disease stage 4 or higher in patients with renal allograft.

**Methods:**

In a cross-sectional study the renal arterial resistive index were obtained in interlobar arteries by Doppler ultrasonography in 78 patients with renal allograft. The stage of chronic kidney disease was determined by the estimated glomerular filtration rate equation.

**Results:**

The median renal arterial resistive index was 0.61 (interquartile range, 0.56 to 0.66). We observed a significant association between renal arterial resistive index above the upper quartile and chronic kidney disease stage 4 or higher (relative risk, 4.64; 95% confidence interval, 1.71 to 12.55; p = 0.003 by Fisher’s exact test). Multivariate logistic regression analysis showed that renal arterial resistive indices (p = 0.02) and time since transplantation (p = 0.04), but not age, gender, or blood pressure were significantly associated with chronic kidney disease stage 4 or higher.

**Conclusion:**

A renal arterial resistive index higher than 0.66 may determine the threshold value of chronic kidney disease stage 4 or higher in patients with renal allograft.

## Introduction

Impaired renal function is frequently observed in patients with renal allograft. Deterioration of renal transplant function is mostly due to chronic allograft nephropathy, which is characterized by chronic interstitial fibrosis, tubular atrophy, vascular occlusive changes, and glomerulosclerosis [1]. Gray-scale ultrasound and Doppler measurements are established noninvasive imaging techniques which have become a routine method for evaluating kidney allografts. Morphologic changes, including the size, parenchymal echogenicity, and corticomedullary differentiation, of the kidney allograft on gray-scale ultrasound may occur in a graft with dysfunction. Furthermore, Doppler measurements may aid in the management of established renal disease by identifying complications in the allograft. Previous studies showed discrepant results whether renal arterial resistive index may predict future events, for example the decrease of 50% or more in creatinine clearance, allograft failure, or death [2]–[7]. However, for clinical practice, measurements of renal arterial resistive index are even more important to highlight the current status of renal allografts. To date there are no data available about the validation of the renal arterial resistive index in terms of stage of kidney disease. Now, we investigated the optimal threshold value of renal arterial resistive index as assessed by Doppler ultrasonography determining chronic kidney disease stage 4 or higher in patients with renal allograft.

## Patients and Methods

### Ethics Statement

All research involving human participants was approved by the local ethics committee (Den Videnskabsetiske Komite for Region Syddanmark, reference number: S-20070059). Informed consent was obtained and all clinical investigation has been conducted according to the principles expressed in the Declaration of Helsinki. Written informed consent was obtained from all patients before entry into the study.

In a cross-sectional study Doppler ultrasonography of the kidney graft was performed in 78 consecutive patients (53 male, 25 female; median age, 54 years, interquartile range, 44 to 66 years) with renal allograft at least three months after transplantation who were seen in our outpatient clinic. Inclusion criteria were the following: 1. Patient with renal allograft at least three months after transplantation. 2. No signs of apparent intercurrent illness. 3. Presence of informed consent. Exclusion criteria were the following: 1. Absence of informed consent. 48 patients (62%) received kidneys from living related donors, 30 patients (38%) from deceased donors, and median time interval since transplantation was 35 months (interquartile range, 10 to 88 months).

At the time of the present investigation all patients were ambulatory and free of intercurrent illness. None of the patients showed signs for acute rejection of kidney allograft. Patient’s history was raised by trained personal using medical records and a standardized questionnaire and comprised personal history and previous history of renal disease and cardiovascular disease. None of the patients had severe tachycardia or bradycardia, which may affect Doppler measurements. Blood pressure was obtained by conventional sphygmomanometric methods on three occasions in a sitting position after a rest of 10 minutes. Phases I and V of the Korotkoff sounds were considered as systolic blood pressure and diastolic blood pressure, respectively.

A glomerular filtration rate less than 30 mL/min/1.73 m^2^ indicated chronic kidney disease stage 4 or higher. Glomerular filtration rate was calculated according to the chronic kidney disease epidemiology collaboration (CKD-EPI) equation [8]. As indicated by Levey et al., variables included in the CKD-EPI equation for estimating log glomerular filtration rate are log serum creatinine (modeled as a two-slope linear spline with sex-specific knots at 62 µmol/L (0.7 mg/dL) in women and 80 µmol/L (0.9 mg/dL) in men), sex, race and age on the natural scale, compared to log serum creatinine without a spline, sex, race and age on the log scale in the MDRD Study equation [8]. In transplant recipients with chronic kidney disease this CKD-EPI formula showed better results than other formulas to estimate glomerular filtration rate [9]. None of the patients with renal allograft was at dialysis treatment at the time of the study.

### Ultrasonographic Determination of the Renal Arterial Resistive Index

A Philips HD-11 XE digital ultrasound machine (Royal Philips Electronics, Amsterdam, Netherlands) with a broadband curved array multifrequency transducer L12-3 with a 2-to-5-MHz extended operating frequency range, field view of 75°, was used for pulsed Doppler measurements. Waveforms were optimized for measurement using the lowest pulse repetition frequency without aliasing, the highest gain without obscuring background noise, and the lowest wall filter [2]. The gray-scale frequency was 5 MHz, the Doppler frequency was set to 2.5 MHz to avoid aliasing. Optimal Doppler gain was set to obtain clear flow waves with minimal background noise. The wall filter was set at 100 Hz. The Doppler sample volume was set at 3 mm. Measurements of the kidney graft were performed with the patients lying in supine position in a quiet room. Valsalva’s maneuver was not performed during Doppler measurements. The gray-scale measurements were performed at the same time as the Doppler measurement of the renal arterial resistive index. The maximal length, width, and depth of the kidney graft were determined. Intrarenal Doppler signals were obtained from two representative interlobar arteries along the border of medullary pyramids. The peak systolic velocity (Vmax) and the minimal enddiastolic velocity (Vmin) were determined, the renal interlobar arterial resistive index was calculated as (Vmax-Vmin)/Vmax, and the results from two measurements were averaged. The peak systolic velocity and the minimal enddiastolic velocity were calculated automatically after manual tracing along the top of the displayed Doppler signals. The Doppler angle was chosen as close to 0° as possible and special care was taken not to compress the kidney. Sonographers were blinded to stage of chronic kidney disease. The intraobserver intrasession variability of duplicate renal arterial resistive index measurements in 78 patients was 2.0% (95% limits of agreement, −18 to 23%). In patients with a renal arterial resistive-index less than 0.66 the intraobserver intrasession variability was 1% (95% limits of agreement, −20 to 22%), whereas in patients with a renal arterial resistive-index higher than 0.66 it was 5% (95% limits of agreement, −14 to 23%). None of the patients had hydronephrosis of grade 2 or higher.

### Statistics

Continuous data are presented as median and interquartile range. Non-parametric Mann-Whitney test was used to detect differences in continuous variables between the groups. Frequency counts were calculated for categorical data such as gender, specific medications, and diagnostic classifications. Differences in these categorical variables between the groups were analyzed by Fisher's exact test. The product limit method of Kaplan and Meier was used to show the fraction of patients presenting with chronic kidney disease stage 4 or higher according to renal arterial resistive index. Curves were compared using the logrank test. Logistic regression analysis was used to determine those variables independently associated with chronic kidney disease stage 4 or higher. The variables tested were renal arterial resistive index, age of renal allograft recipient, time since transplantation, systolic blood pressure, diastolic blood pressure, and pulse pressure. For multivariate analysis, the effect of multiple variables on the presence of chronic kidney disease stage 4 or higher was evaluated in 78 patients with stepwise forward regression analysis (with p = 0.10 as the threshold level of significance for the removal of the variable from analysis and p = 0.05 as the threshold for entry into the model). Data were analyzed using GraphPad prism software (version 5.0, GraphPad Software, San Diego, CA, USA) and SPSS for windows (version 15.0; SPSS, Chicago, IL, USA). All statistical tests were two-sided. Two-sided p-values less than 0.05 were considered to indicate statistical significance.

## Results

Doppler ultrasonography of kidney graft was performed in 78 patients with renal allograft. 53 (68%) transplant recipients were male, and 25 (32%) were female. Median age of recipients was 54 years (interquartile range, 44 to 66 years). The cause of chronic kidney disease was hypertensive nephropathy in 7 patients (9%), diabetes mellitus in 9 patients (11%), glomerulonephritis in 24 patients (31%), polycystic kidney disease in 13 patients (17%), reflux nephropathy in 4 patients (5%), and others/unknown in 21 patients (27%). Median time on dialysis (dialysis vintage) was 20 months (interquartile range, 7 to 36 months).

48 patients (62%) received kidneys from living related donors, and 30 patients (38%) from deceased donors. Median donor age was 49 years (interquartile range, 42 to 57 years). Median time interval since transplantation was 35 months (interquartile range, 10 to 88 months). The number of patients with renal allograft presenting with chronic kidney disease stage 1 (glomerular filtration rate ≥90 mL/min per 1.73 m^2^), stage 2 (glomerular filtration rate between 60 to 89 mL/min per 1.73 m^2^), stage 3 (glomerular filtration rate between 30 and 59 mL/min per 1.73 m^2^), stage 4 (glomerular filtration rate between 15 and 29 mL/min per 1.73 m^2^), and stage 5 (glomerular filtration rate <15 mL/min per 1.73 m^2^) were 5 (6%), 22 (28%), 38 (49%), 12 (16%), 1 (1%), respectively.

For all patients the median renal arterial resistive index was 0.61 (interquartile range, 0.56 to 0.66). The median renal arterial resistive index was not significantly different in male patients (0.61; interquartile range, 0.56 to 0.66), compared to female patients (0.60; interquartile range, 0.55 to 0.68; p = 0.80). Patients were stratified according to renal arterial resistive index below or above the upper quartile. Using receiver-operating-characteristic curve this threshold showed a specificity of 85% and sensitivity of 62%. The clinical and biochemical characteristics of patients and their allograft are shown in **Table 1** and **Table 2**. Patients with renal arterial resistive index above the upper quartile were older, had lower glomerular filtration rate and higher blood urea nitrogen levels.

**Table 1:** Clinical characteristics of patients with renal allograft.

Characteristic	RI <0.66	RI ≥0.66	p-value
Age (years)	52 (43 to 62)	64 (49 to 70)	0.01
Gender			
male, number (%)	43 (74)	10 (50)	
female, number (%)	15 (26)	10 (50)	
Number of patients with a history of more than 1 transplantation (%)	10 (17)	3 (15)	n.s.
Duration of dialysis before transplantation (months)	16 (2 to 36)	33 (14 to 36)	n.s.
Body weight (kg)	87.0 (77.4 to 93.2)	71.2 (58.0 to 84.2)	0.01
Body mass index (kg/m^2^)	27.4 (24.9 to 30.5)	25.1 (22.5 to 28.1)	n.s.
Systolic blood pressure (mmHg)	136 (130 to 145)	137 (135 to 143)	n.s.
Diastolic blood pressure (mmHg)	81 (78 to 85)	78 (74 to 80)	n.s.
Pulse pressure (mmHg)	55 (50 to 65)	59 (51 to 67)	n.s.
Immunosuppressive medication, number (%)			
Steroids	12 (21)	6 (30)	n.s.
Cyclosporine or tacrolimus	57 (98)	17 (85)	n.s.
Mycophenolate mofetil	52 (90)	17 (85)	n.s.
Other	4 (7)	4 (20)	n.s.
Antihypertensive medication, number (%)			
Calciumantagonists	38 (66)	11 (55)	n.s.
Angiotensin-converting-enzyme inhibitors or Angiotensin AT1-receptor antagonists	27 (46)	10 (50)	n.s.
Betablocker	28 (48)	12 (60)	n.s.
Number of patients with history of cytomegalovirus infection (%)	12 (21)	3 (15)	n.s.
Number of patients with rejection episodes (%)	13 (22)	3 (15)	n.s.
Smoking, number (%)	13 (22)	1 (5)	n.s.
Other diseases, number (%)			
Diabetes mellitus	12 (21)	1 (5)	n.s.
Hypertension	46 (79)	18 (90)	n.s.
History of cardiovascular events	21 (36)	5 (25)	n.s.
Age of donor (years)	48 (42 to 57)	50 (47 to 61)	n.s.
Delayed graft function >6 days, number (%)	2 (3)	2 (10)	n.s.
Living kidney donor, number (%)	39 (67)	9 (45)	n.s.
Time since transplantation (months)	33 (10 to 78)	35 (14 to 96)	n.s.

Patients were stratified according to renal arterial resistive index (RI) below or above the upper quartile threshold value of 0.66. Continuous data are presented as median (interquartile range). Non-parametric Mann-Whitney test was used to detect differences in continuous variables between the groups. Differences in categorical variables between the groups were analyzed by Fisher's exact test.

**Table 2:** Biochemical characteristics of patients with renal allograft.

Characteristic	RI <0.66	RI ≥0.66	p-value
Hemoglobin (mmol/L)	8.3 (7.5 to 9.0)	7.7 (7.4 to 8.7)	n.s.
Glomerular filtration rate (CKD-EPI) (mL/min per 1.73 m^2^)	53 (39 to 70)	43 (26 to 63)	0.05
Blood urea nitrogen (mmol/L)	8.7 (6.6 to 11.3)	12.4 (8.9 to 17.1)	0.05
Serum calcium (mmol/L)	1.28 (1.22 to 1.32)	1.28 (1.22 to 1.35)	n.s.
Serum phosphate (mmol/L)	0.91 (0.74 to 1.01)	0.92 (0.77 to 0.99)	n.s.
Total cholesterol (mmol/L)	5.2 (4.6 to 6.0)	6.0 (4.5 to 6.5)	n.s.
C-reactive protein (mg/L)	3 (2 to 7)	2 (1 to 4)	n.s.

Patients were stratified according to renal arterial resistive index (RI) below or above the upper quartile (0.66). Continuous data are presented as median (interquartile range). Non-parametric Mann-Whitney test was used to detect differences in continuous variables between the groups.

**Figure 1:**
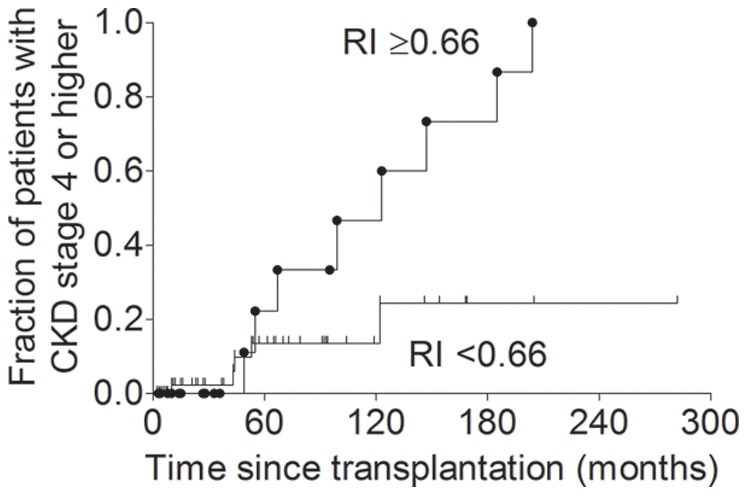
Kaplan-Meier estimates of the fraction of patients presenting with chronic kidney disease stage 4 or higher according to renal arterial resistive index. Patients were stratified according to renal arterial resistive index (RI) below or above the upper quartile, i.e. 0.66. Relative risk, 4.64; 95% confidence interval, 1.71 to 12.55; p = 0.003.

We observed a significant association between renal arterial resistive index above the upper quartile and chronic kidney disease stage 4 or higher (relative risk, 4.64; 95% confidence interval, 1.71 to 12.55; p = 0.003 by Fisher’s exact test). **Figure 1** shows Kaplan-Meier estimates of the fraction of patients presenting with chronic kidney disease stage 4 or higher according to renal arterial resistive index (Chi-square 5.57; p = 0.02 by Log-rank (Mantel-Cox) Test).

Univariate logistic regression analysis showed that renal arterial resistive index (p = 0.008), time since transplantation (p = 0.018), and pulse pressure (p = 0.021) were significantly associated with chronic kidney disease stage 4 or higher, whereas age, gender, systolic and diastolic blood pressure where not associated with chronic kidney disease stage 4 or higher (each p>0.05). Using multivariate logistic regression analysis we observed that renal arterial resistive index (p = 0.02) and time since transplantation (p = 0.04), but not age, gender, systolic blood pressure, diastolic blood pressure, nor pulse pressure were significantly associated with chronic kidney disease stage 4 or higher.

## Discussion

In the present study we show that a renal arterial resistive index higher than 0.66 in the kidney allograft allows optimal distinction of patients with chronic kidney disease stage 4 or higher from the other patients with renal allograft. Normal values for renal arterial resistive index in healthy subjects have been reported previously. In 135 healthy subjects without preexisting disease (median age, 37 years) the mean renal arterial resistive index was 0.59 [10]. Measurements in 34 living kidney donors showed that the resistive index in the remnant kidney of healthy donors remained stable during follow up [11].

The renal arterial resistive index was advanced as a useful parameter for quantifying the alterations in the kidney that may occur with renal disease. However, the origin of resistive index and the causes of increased resistive index in kidney diseases are not completely evaluated. Experiments on isolated perfused rabbit kidneys revealed that the renal arterial resistive index increased with decreases in the cross-sectional area of the distal arterial bed [12]. Moreover, the renal arterial resistive index has been positively correlated with histopathologic changes in the diseased kidney, i.e. with the amount of glomerular sclerosis and interstitial fibrosis in kidney biopsies [13]. Ikee et al. showed that, both, histopathologic parameters and histological signs of atherosclerosis in kidney vessels showed statistically significant correlations with renal arterial resistive index [14]. Therefore, renal scaring with vascular wall medial thickening with frequent arteriolar hyaline deposits, varying degrees of intimal fibrosis and focal glomerular ischemic changes, proportional tubular atrophy and interstitial fibrosis may cause reduced vessel area and finally increased renal arterial resistive index [15]. Alterations in the kidney tissue and in the vasculature may contribute to changes of resistive index with decreasing kidney function.

Conflicting data have been reported concerning the use of renal arterial resistive index to predict future events, i.e. loss of renal allografts and deaths. A cohort study by Radermacher et al. showed that a renal arterial resistive index of 0.80 or higher measured at least three months after transplantation was predictive of a combined endpoint including a decrease of 50% or more in creatinine clearance, allograft failure, or death [2]. DeVries et al. showed that the renal vascular resistive index, which was based on blood pressure and renal blood flow, was a prominent risk marker for recipient mortality and death-censored graft loss [3]. A recent study by Krol et al. using intraoperative transit time flowmetry showed that patients with renal arterial resistive index of 0.57 or higher had significantly lower estimated glomerular filtration rate 48 months after transplantation [4]. McArthur et al. showed that the resistive index obtained within 1 week after transplantation was an independent predictor of death-censored transplant survival [5]. However, several studies reported contradictory results. Loock et al. reported that neither 4-month nor 1-year renal arterial resistive index predicted loss of kidney allografts [6]. The study by Gerhart et al. did not confirm that a renal arterial resistive index higher than 0.80 may predict event-free transplant survival [7]. Therefore, it is unknown whether or not the renal arterial resistive index may predict future events. A recent study showed that determination of the resistive index seems to be a promising tool to assess the risk of acute kidney injury even in critically ill patients [16]. Furthermore a recent study Doi et al. showed that the renal resistive index can predict outcome particularly in hypertensive patients with chronic kidney disease [17]. This study may indicate the clinical need to determine the renal resistive index in the native kidneys from patients with chronic kidney disease, too.

In our cohort the percentage of living kidney donors (62%) was much higher compared to previous reports. The cohort reported by Radermacher et al. contained only 7% living kidney donors, and in the study by Gerhart et al. no living kidney donors were reported [2], [7]. The determination of the resistive index in patients with living related kidney donors and kidneys from deceased donors may show differences. It is known that kidneys from deceased donors are prone to alterations to due to longer cold ischemic time and particularly in cadaveric donors of older age to age-associated diseases. Therefore the effects of transplantation may be more easily evaluated in patients with living related kidney donors. These circumstances may explain that only renal arterial resistive index and time since transplantation were significantly associated with chronic kidney disease stage 4 or higher. However, the higher number of patients with living related donors may be a limitation of the present study. The observed threshold for the resistive index should be reassessed in a study determining the resistive index only in kidneys from deceased donors.

In summary, a renal arterial resistive index higher than 0.66 may determine chronic kidney disease stage 4 or higher in patients with renal allograft.
